# Blocking drug efflux mechanisms facilitate genome engineering process in hypercellulolytic fungus, *Penicillium funiculosum* NCIM1228

**DOI:** 10.1186/s13068-021-01883-4

**Published:** 2021-01-25

**Authors:** Anmoldeep Randhawa, Nandita Pasari, Tulika Sinha, Mayank Gupta, Anju M. Nair, Olusola A. Ogunyewo, Sandhya Verma, Praveen Kumar Verma, Syed Shams Yazdani

**Affiliations:** 1 Microbial Engineering Group, International Centre for Genetic Engineering and Biotechnology, New Delhi, 110067 India; 2 DBT-ICGEB Centre for Advanced Bioenergy Research, International Centre for Genetic Engineering and Biotechnology, New Delhi, 110067 India; 3 National Institute of Plant Genome Research, New Delhi, 110067 India

**Keywords:** Drug tolerance, *Agrobacterium*-mediated transformation, CRISPR/Cas9, Genome modification, Cellobiohydrolase I

## Abstract

**Background:**

*Penicillium funiculosum* NCIM1228 is a non-model filamentous fungus that produces high-quality secretome for lignocellulosic biomass saccharification. Despite having desirable traits to be an industrial workhorse, *P. funiculosum* has been underestimated due to a lack of reliable genetic engineering tools. Tolerance towards common fungal antibiotics had been one of the major hindrances towards development of reliable transformation tools against the non-model fungi. In this study, we sought to understand the mechanism of drug tolerance of *P. funiculosum* and the provision to counter it. We then attempted to identify a robust method of transformation for genome engineering of this fungus.

**Results:**

*Penicillium funiculosum* showed a high degree of drug tolerance towards hygromycin, zeocin and nourseothricin, thereby hindering their use as selectable markers to obtain recombinant transformants. Transcriptome analysis suggested a high level expression of efflux pumps belonging to ABC and MFS family, especially when complex carbon was used in growth media. Antibiotic selection medium was optimized using a combination of efflux pump inhibitors and suitable carbon source to prevent drug tolerability. Protoplast-mediated and *Agrobacterium*-mediated transformation were attempted for identifying efficiencies of linear and circular DNA in performing genetic manipulation. After finding Ti-plasmid-based *Agrobacterium*-mediated transformation more suitable for *P. funiculosum*, we improvised the system to achieve random and homologous recombination-based gene integration and deletion, respectively. We found single-copy random integration of the T-DNA cassette and could achieve 60% efficiency in homologous recombination-based gene deletions. A faster, plasmid-free, and protoplast-based CRISPR/Cas9 gene-editing system was also developed for *P. funiculosum*. To show its utility in *P. funiculosum*, we deleted the gene coding for the most abundant cellulase Cellobiohydrolase I (CBH1) using a pair of sgRNA directed towards both ends of *cbh1* open reading frame. Functional analysis of ∆*cbh1 *strain revealed its essentiality for the cellulolytic trait of *P. funiculosum* secretome.

**Conclusions:**

In this study, we addressed drug tolerability of *P. funiculosum* and developed an optimized toolkit for its genome modification. Hence, we set the foundation for gene function analysis and further genetic improvements of *P. funiculosum* using both traditional and advanced methods.

## Background

Bioprospecting often expedites discovery of non-model microorganisms for addressing the problems left unrequited by traditional industrial workhorses. Cost-effective lignocellulolytic enzyme development is a field of study that has been benefitted extensively by bioprospecting research. Our probe identified *Penicillium funiculosum* NCIM1228 whose secretome demonstrated exceptional saccharification capabilities [1]. NCIM1228 is non-pathogenic, has micro-diversity of extracellular enzyme genes, possesses exceptional secretion capabilities and exhibits experimental tractability [2]. Although the organism has evolved to grow on recalcitrant cellulosic biomass, it cannot be utilized directly as cell factory to produce ‘most performing’ secretome. Genetic reprogramming of biosynthesis pathways is crucial to develop it into an industrial workhorse. Development of molecular toolkit is therefore an essential step to expand the biotechnological potential of *P. funiculosum*.

Attempts were made previously to improve the quality of secretome of *P. funiculosum*, but the genetic modifications were achieved by exposure to physical (UV/gamma radiations) and chemical mutagens [3]. Random approaches like these prove limiting for intensive strain engineering and pathway rewiring at genome level. Targeted integration was attempted by polyethylene glycol (PEG)-mediated transformation of linearized deletion cassettes into *P. funiculosum* protoplasts [4]. A deletion cassette for gene of interest was prepared by utilizing approximately 1–2 kb homologous sequence on either side of the selectable marker cassette. However, recombination frequency varied with gene locus as ectopic recombination being prominent than homologous recombination in filamentous fungi. Besides, the use of antibiotics for selecting the transformants in filamentous fungi is a rigorous process because of high percentage of false-positive transformants [5]. A recent study described a marker-less gene deletion system where linearized integrative plasmids, having *pyr4* as selection marker, were used for increasing the efficiency of homologous recombination [6].

This study was sought to develop genetic tools and methodologies related to genome editing and modification in *P. funiculosum* NCIM1228. The phenomenon of drug tolerance in NCIM1228 was determined by transcriptomic data analysis, and selection medium was customized accordingly to achieve absolute sensitivity of *P. funiculosum* NCIM1228 towards three antibiotics (hygromycin, zeocin and nourseothricin) [7]. Antibiotic resistance marker cassettes were constructed using constitutive promoters and terminators from *P. funiculosum* NCIM1228 and tested for their pertinency. *Agrobacterium*-mediated transformation method was optimized for gene deletions in *P. funiculosum* NCIM1228. Additionally, a faster genome engineering technique was also developed by using plasmid-free CRISPR/Cas9 system. Together, these new genetic tools provide the foundation to broaden the scope of *P. funiculosum* as an improved industrial organism. Cellobiohydrolase I is the most abundant cellulase present in the cellulolytic secretome of NCIM1228 [1]. Using abovementioned tools, *cbh1* gene deletion strain was created and functional analysis studies were conducted to understand the impact of *cbh1* deletion on growth and secretome of NCIM1228.

## Results

### Design of selection medium for improved drug sensitivity

**Fig. 1:**
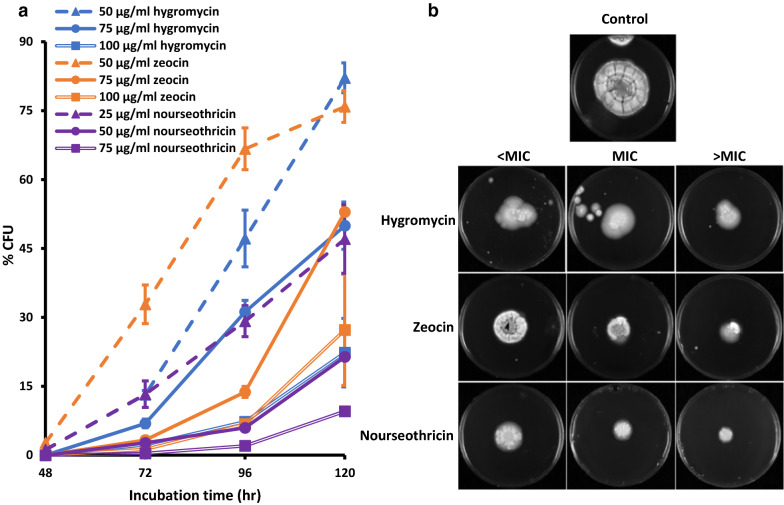
Drug tolerance in *P. funiculosum* NCIM1228. **a** 100 µl of 10^4^ spores/ml were plated on PD agar having each of the three antibiotics at below MIC, MIC and above MIC level, and colonies were counted at different time intervals. The graph shows the average values of three PD agar plates for each drug concentration. **b** Equal number of NCIM1228 spores were spotted on potato dextrose plates having antibiotics at MIC, < MIC and > MIC. Pictures were taken after 5 days of incubation

Foremost requirement of transformation is an efficient screening system to allow selective survival and growth of desired transformants. Genomic integration of antibiotic resistance cassettes allows for selection of mutants under antibiotic selective pressure. Blast search of *P. funiculosum* NCIM1228 genome draft sequence could not retrieve any gene sequence corresponding to resistance genes for three most common antibiotics used as selection markers, namely, hygromycin, zeocin and nourseothricin. To determine the inhibitory concentration of these antibiotics for *P. funiculosum* NCIM1228, 10^4^ spores were plated on PD agar having antibiotics with concentration ranging from 25 µg/ml to 100 µg/ml. *P. funiculosum* NCIM1228 was found susceptible to hygromycin and zeocin at minimal inhibitory concentration (MIC) of 75 µg/ml media and nourseothricin at 50 µg/ml in PD agar after two days of incubation (Additional file 1: Table S2 and Fig. S1a). However, *P. funiculosum* NCIM1228 takes 7–14 days to form a fungal colony [8] and transformants would require the same time frame to appear in the presence of antibiotics. We, thus, determined the percentage of NCIM1228 spores that could develop into colony forming units (CFU) in the presence of three antibiotics at below MIC level (< MIC), MIC level and above MIC level (> MIC) of drug concentrations for prolonged periods of time (Fig. 1a). In case of hygromycin and zeocin, > 75% of the spores were able to form CFU after 5 days of incubation at drug concentration below MIC (i.e. at 50 µg/ml), whereas more than 50% of the spores could form colonies at MIC (i.e. at 75 µg/ml). NCIM1228 was found more susceptible towards nourseothricin, wherein > 40% of the cells could form CFU below MIC (i.e. at 25 µg/ml) and CFU reduced to 20% of the total spore count at MIC (i.e. at 50 µg/ml). However, the CFU percentage dropped at > MIC for all three antibiotics, yet we could not achieve complete inhibition of mycelial growth (Fig. 1a). We next determined the colony formation by NCIM1228 spores on PD agar plates having antibiotics at all the three concentrations (Fig. 1b). Colony formation was found at all concentrations for all three drugs after five days of incubation; however, the colony size reduced with increasing drug concentration (Fig. 1b). Recent studies have established the phenomenon of drug tolerance in fungal pathogens which is different from drug resistance. Drug tolerance has been defined as the ability of a drug-susceptible fungal strain to grow at drug concentrations above the minimum inhibitory concentration (MIC) [9]. Mycelial growth observed at MIC and above MIC drug concentrations after 48 h of incubation suggested that *P. funiculosum* exhibited a substantial degree of tolerance towards glycopeptide (e.g. zeocin) and aminoglycoside (e.g. hygromycin and nourseothricin) classes of antibiotics.

**Fig. 2:**
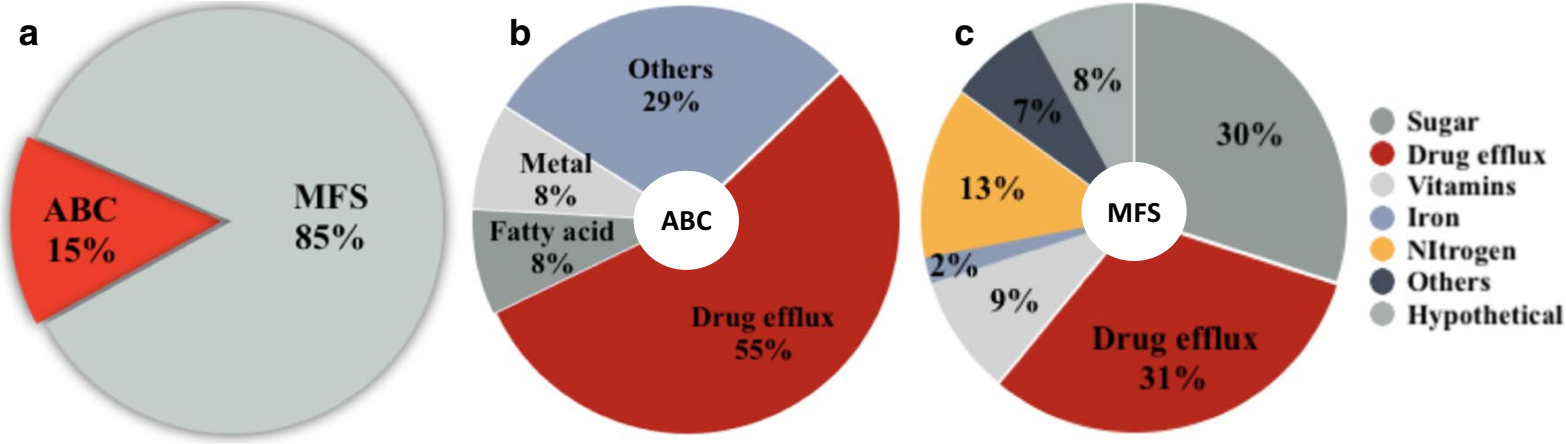
Drug transporters in *P. funiculosum* NCIM1228 genome. Pie chart representing **a** the percentage profile of ABC and MFS transporters among total transporters present in the NCIM1228 genome, **b** the functional distribution of ABC transporters and **c** MFS transporters

The first challenge to genetic transformation of NCIM1228 was to determine growth conditions that could suppress drug tolerance and enhance sensitivity towards selection drugs. Drug tolerance in fungal pathogens has been linked to drug efflux pumps that maintain low intracellular levels of drug, resulting in increased viability even at higher concentrations of drug [10]. InterProScan of draft genome sequence of *P. funiculosum* identified a total of 430 membrane transporter genes. Out of these, 62 (15%) transporter genes belonged to ATP-Binding Cassette (ABC) superfamily and 368 (85%) genes were predicted to code for multi-facilitator superfamily (MFS) transporters (Fig. 2a) [11]. Role of ABC transporters has been implicated in heavy metal and drug resistance in plant and fungi [11]. In case of *P. funiculosum*, 34 ABC transporters were predicted to function as drug efflux pumps, representing predominant fraction (55%) among ABC transporters (Fig. 2b), while 5 (8%) each were putative fatty acid and metal ion transporters, including iron, and the remaining 18 (29%) of ABC transporters were predicted to transport diverse biomolecules. MFS transporters use the electrochemical proton-motive force to power drug efflux. Here, 201 (55%) of MFS transporter genes were anticipated to be involved in the transport of carbon and nitrogen sources, metal ions, minerals and vitamins. 119 (30%) of the MFS transporter genes were projected as drug efflux pumps and the remaining 52 (15%) genes were either hypothetical or had unknown functions (Fig. 2c). In summary, more than half of the ABC transporter genes and about one-third of MFS transporter genes code for drug efflux pumps, and comprehending qualitative and quantitative expression profiling of these drug transporter genes, with a focus on abolishing drug tolerance, thus, was the key to find the best-fit selection medium for NCIM1228.

**Fig. 3:**
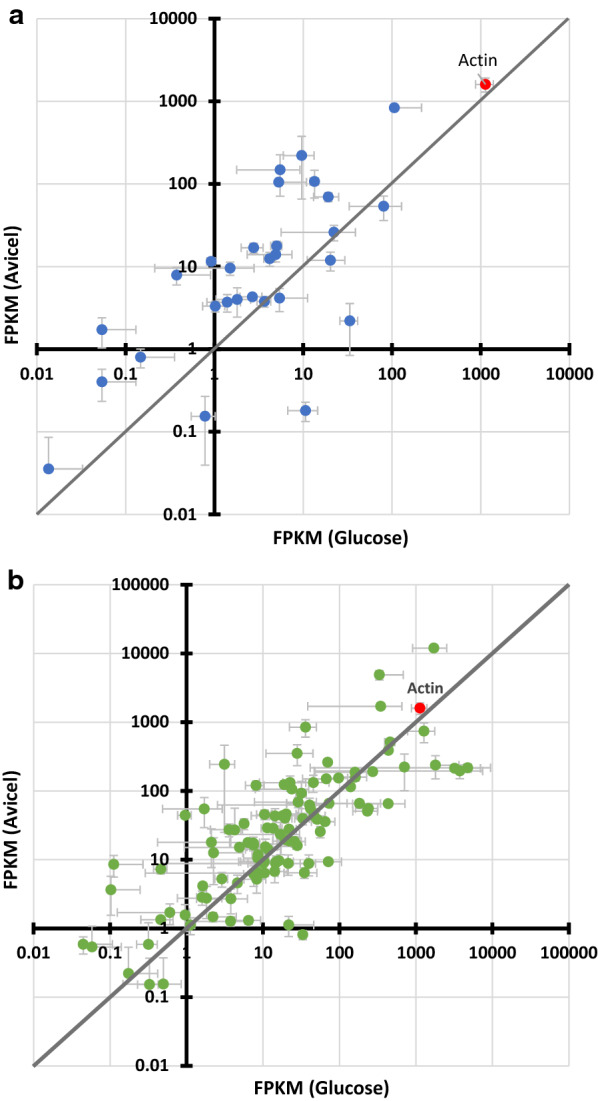
Expression level of transcripts of drug transporter genes in the presence of glucose and Avicel. **a** Scatter plot representing non-centred expression profile of ABC drug efflux pump genes in the presence of glucose and Avicel. 5 ABC transporter genes did not exhibit expression in either or both carbon sources and thus excluded from logarithmic plot. **b** Scatter plot representing non-centred expression profile of MFS drug efflux pump genes in the presence of glucose and Avicel. 11 MFS transporter genes did not exhibit expression in either or both carbon sources and thus excluded from logarithmic plot. The data represent average and standard deviation of two independent biological replicates

We undertook a directed transcriptomic approach to study the role of drug efflux transporters in drug tolerance displayed by *P. funiculosum* NCIM1228*.* Since carbon is a major constituent of any growth medium, we were interested to know if the expression of drug transporters is carbon source dependent. For that reason, we carried out transcriptomic study in the presence of fermentable sugar (glucose) and recalcitrant cellulose (Avicel). Transcriptomic data were generated by using Illumina RNA-seq technology and expression profile of each gene was normalized against the whole data size to calculate FPKM (fragments per kilobase of exon model per million mapped reads) (Fig. 3). The FPKM values of gene encoding actin, a highly expressed housekeeping gene, served as a bench mark to judge the level of expression of drug transporter genes (Fig. 3). Among 34 ABC transporter genes predicted to function as drug efflux pumps, 5 showed negligible expression and none of the genes showed expression levels similar to that of actin under both carbon conditions (Fig. 3a). Some of the genes that showed relatively higher expression included genes for multidrug resistance protein 1 (Mdr1) and multidrug resistance protein 2 (Mdr2). On the other hand, many MFS drug transporter genes showed equivalent mRNA abundance as that of actin under both carbon conditions and could be major contributors towards drug tolerance exhibited by NCIM1228 (Fig. 3b); 11 out of 119 MFS efflux pump genes did not exhibit expression in either or both carbon sources.

We also observed variable expression pattern of ABC and MFS drug transporter genes in response to glucose and Avicel, which suggested their dependency on the type of carbon source used for fungal growth. Of the 29 ABC drug transporter genes expressed, 23 genes (79%) showed higher expression in Avicel as compared to glucose (Fig. 3a). The dependency was little less prominent in case of MFS drug transporter genes where 60 out of 109 expressed genes (55%) showed higher expression in Avicel (Fig. 3b). These arrays of ABC as well as MFS drug efflux pumps detected in our transcriptomic analysis could possibly explain the observed drug tolerance in *P. funiculosum* NCIM1228.

**Fig. 4:**
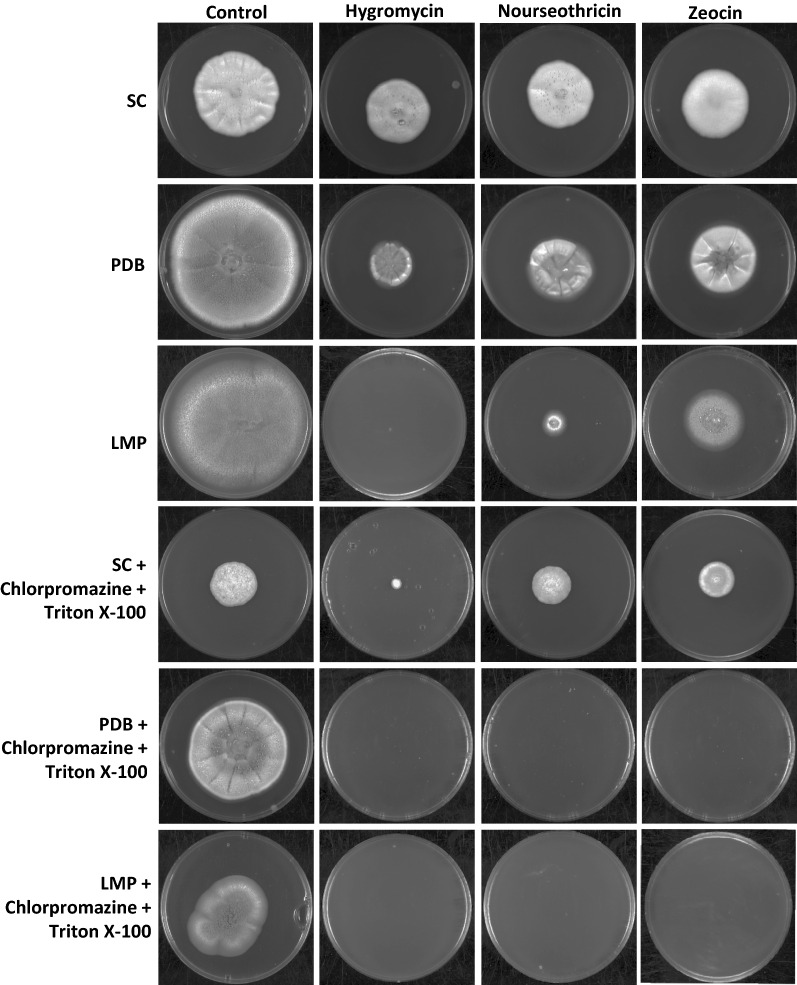
Sensitivity of *P. funiculosum* NCIM1228 towards antibiotics on different growth mediums. Equal number of spores was spotted on growth media and incubated at 30 °C for 7 days. 50 µg/ml of hygromycin, zeocin and nourseothricin were added to the growth media with/without 0.1 mM chlorpromazine and 0.01% Triton X-100

Inference derived from transcriptome was further applied to identify a suitable selection medium that leads to less expression of drug efflux pumps and combats the issue of drug tolerance to avoid false positives during transformation. We avoided any media with polymeric sugars like cellulose, instead focused on medium having free sugars. We tested three media, potato dextrose (PD) agar, yeast nitrogen base with 2% glucose (SC) agar and modified low malt extract soya peptone (LMP) agar and evaluated the susceptibility of *P. funiculosum* towards three antibiotics, namely, hygromycin, zeocin, and nourseothricin at 50 µg/ml after 7 days of incubation (Fig. 4). *P. funiculosum* showed varying tolerance to these drugs on the three media and level of drug tolerance was in the order of SC > PD > LMP. Additionally, morphotype displayed by *P. funiculosum* NCIM1228 was distinct on all three growth media. Colonies formed on SC agar were white, tufted with smooth floccose and exhibited higher ratio of aerial hyphae with minimal agar invasion. Moreover, colonies did not cover the entire available surface on the agar plates and never sporulated. The presence of antibiotics in YNB agar marginally affected the colony size of *P. funiculosum* NCIM1228; however, colonies were no longer tufted. NCIM1228 formed flat, grey and highly embedded colonies on PD agar with grey spores. The presence of antibiotics could only reduce the size of colonies on PD agar. Colony morphology on LMP agar was unique in terms of scarce mycelial growth, heavy sporulation and green-coloured spores. Colony size reduction was maximum on LMP agar in the presence of antibiotics. According to the transcriptomic data, achieving high level tolerance to all three drugs required abolishing the expression of 46 most significantly expressed transporter genes, which seemed impractical. Therefore, a more rationalized approach was to use chemical inhibitors against drug efflux proteins. Since MFS transporters are more abundant and diverse in their role, using chemical inhibitors against them in the selection medium would be deleterious for overall growth of fungi. However, drug export through electrochemical gradient could be counteracted by increasing the membrane permeability by the use of non-ionic detergents, like Triton X-100. Further, using chemical inhibitors against ABC transporters could be beneficial as they are less in number as well as functionally less diverse than MFS transporters. Earlier studies on *Aspergillus niger* protoplasts have shown that chlorpromazine along with Triton X-100 was more effective than other ABC drug transporter inhibitors [12]; we added 0.1 mM chlorpromazine and 0.01% Triton X-100 in the three growth media and found marginal reduction in colony size of NCIM1228 (Fig. 4). When antibiotics were added along with chlorpromazine and Triton X-100, *P. funiculosum* gained complete sensitivity for all three antibiotics in PD agar and LMP agar; however, it was still found to be tolerant towards all the three drugs in SC agar after 7 days of incubation (Fig. 4). Further, no growth was observed in the presence of hygromycin and nourseothricin even after 14 days of incubation on PD and LMP agar supplemented with chlorpromazine and Triton X-100, though marginal drug tolerance was exhibited by NCIM1228 on zeocin plates (Additional file 1: Fig. S2). For NCIM1228, LMP agar emerged as the most effective medium for using the drugs as selection markers for longer periods of incubation. Further, chlorpromazine and Triton X-100 in combination, and not alone, were effective in supporting the drug tolerance (Additional file 1: Fig. S3). Hygromycin and nourseothricin were found to be equally potent drugs among the three drugs tested for *P. funiculosum*. We used LMP agar supplemented with 0.1 mM chlorpromazine and 0.01% Triton X-100 along with appropriate antibiotic for standardization of transformation protocols and referred it as optimized selection medium.

### Mode of transformation for random integration in fungal genome

Two methods are popular for DNA transformation in filamentous fungi, i.e. protoplast based and *Agrobacterium*-mediated transformation. Both these methods were attempted for transformation of *P. funiculosum*. For protoplast-mediated method, a synthetic vector pAN-egfp containing bleomycin resistance gene and reporter *egfp* gene was used for PEG-mediated protoplast transformation of *P. funiculosum* (Additional file 1: Fig. S4a). The transformation was carried out with approximately 10^7^ protoplast cells and 5 µg pAN-egfp linearized DNA. Repeated transformations under similar conditions gave us about 10 transformants per round. However, passaging for 5 times in the presence of zeocin resulted in the loss of viability of the transformants on optimized selection medium. It might be due to the loss of transformed cassette after few generations.

**Fig. 5:**
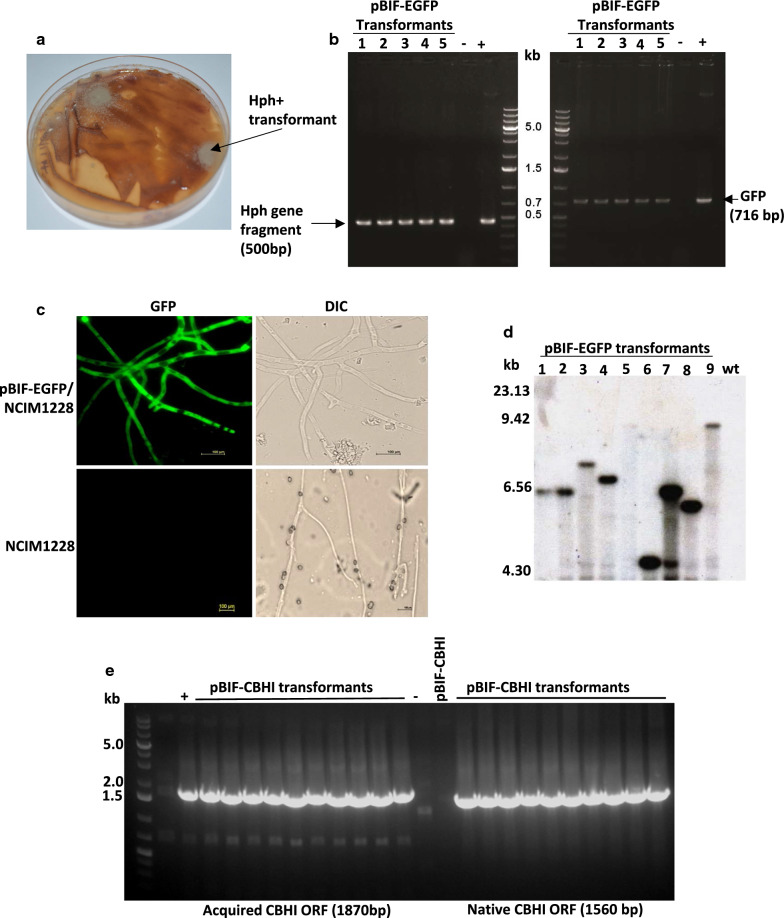
*Agrobacterium*-mediated transformation of pBIF-egfp in *P. funiculosum* NCIM1228. **a** Hygromycin-resistant transformants on LMP hygromycin selection plate with Chlorpromazine and Triton X-100. **b** PCR confirmation of transformants. Left panel shows the amplification of 500 bp fragment of hygromycin cassette and right panel shows amplification of reporter gene EGFP from genomic DNA of hygromycin-resistant transformants, with no amplification in lane with NCIM1228 genomic DNA (-ve control). + ve lane showed amplification from pBIF-egfp construct. **c** Fluorescence microscopy of pBIF-egfp transformants showing GFP fluorescence, NCIM1228 was taken as negative control. **d** Southern blot of pBIF-egfp transformants showing integration of T-DNA having *egfp* gene. E-GFP PCR amplified fragment was used as probe. NCIM1228 (WT) was taken as negative control. **e** PCR-based detection of two copies of *cbh1* gene, one native and second recombinant, in hygromycin-resistant pBIF-CBH1 transformants. Left panel shows 1870 bp amplification of acquired *cbh1* gene under GPD promoter using hygromycin cassette internal primer and *cbh1* gene internal reverse primer. NCIM1228 was taken as negative control and pBIF-EGFP was taken as positive control. Right panel shows 1560 bp amplification of innate copy of *cbh1* gene under native promoter. pBIF-egfp was taken as negative control showing no amplification

Next, *Agrobacterium*-mediated method of transformation was attempted with pBIF-egfp plasmid having hygromycin phosphotransferase gene (*hph*) as selection marker and *egfp* as reporter gene [13]. The induced *Agrobacterium* harbouring the plasmid pBIF-egfp was co-cultivated with *P. funiculosum* spores on IM agar plates containing cellophane discs and the membrane discs were placed on the LMP plates having hygromycin at 50 µg/ml for selection. The frequency of transformation here was observed to be relatively higher, i.e. > 100 transformants/10^6^ spores appeared within 5–10 days of incubation at 30 °C (Fig. 5a). The transformants were transferred to fresh selection plates and passaged multiple times and were found to be stable. We confirmed the presence of T-DNA by PCR of 500 bp internal region of the *hph* gene along with 716 bp *egfp* ORF (Fig. 5b). We also checked the expression of the *egfp* reporter gene in the *P. funiculosum* transformants by fluorescence microscopy (Fig. 5c). The EGFP fluorescence was observed in all the antibiotic-resistant fungal colonies, but the level of fluorescence was variable (Additional file 1: Fig. S4b) perhaps due to differences in the sites of integration as well as integrated copies of *egfp* cassette (Additional file 1: Fig. S4b). GFP expression was stable even in the absence of selection pressure, indicating the mitotic stability of the T- DNA region of the construct. We checked the copy number of pBIF-egfp T-DNA in *P. funiculosum* by Southern hybridization using PCR-amplified *egfp* as probes [14] (Additional file 1: Fig. S4c). We found all the tested transformants, except Transformant 7, having a single gene integration (Fig. 5d). Transformant 7 had two copies of *egfp* cassette and also exhibited the highest GFP fluorescence intensity (Additional file 1: Fig. S4b).

We proceeded to use this method to integrate an additional copy of an important native gene to obtain its over-expression. Cellobiohydrolase I (PfCBH1) was identified as a key cellulase secreted by NCIM1228 under cellulase-inducible conditions and was a major factor for exceptional cellulolytic activity of NCIM1228 secretome [15]. With intent to increase its expression in NCIM1228, *gfp* ORF in pBIF-egfp was replaced with native *cbh1* gene of *P. funiculosum* NCIM1228 and the resultant plasmid was used to transform NCIM1228. The transformants were screened for an additional copy of the *cbh1* gene by PCR. Since the two copies of *cbh1* gene would be under two different promoters in transformants (native *cbh1* and *gpd* promoter), we confirmed it by amplifying DNA fragments using one internal primer of *cbh1* gene and other primer from either of the promoter. NCIM1228 showed amplification only for the native *cbh1* copy with native promoter, whereas transformants showed amplification for both *cbh1* gene copies (Fig. 5e). The Southern blotting of genomic DNA of hygromycin-resistant transformants with hygromycin gene probe showed single-copy integration of the cassette (Additional file 1: Fig. S4d, S4e). RNA transcripts of *cbh1* in the transformants were quantified by real-time PCR and showed > fivefold increase as compared to NCIM1228 (Additional file 1: Fig. S4f). We, thus, demonstrated single-copy random gene integration of native as well as heterologous gene in *P. funiculosum* NCIM1228 using *Agrobacterium*-mediated transformation.

### Homologous recombination via *Agrobacterium*-mediated transformation

Homologous recombination offers a targeted approach to create deletions, mutations and insertions in the genome. Since protoplast-mediated method of transformation failed in *P. funiculosum*, we attempted to carry out homologous recombination by utilizing *Agrobacterium*-mediated binary vector-based transformation. For facilitating deletion of the gene via homologous recombination, we chemically synthesized hygromycin and zeocin resistance cassettes under NCIM1228 *trpC* promoter and terminator for creating two independent disruption cassettes (Fig. S5a and S5b). We then made attempt to disrupt cellobiohydrolase I (*cbh1*) gene in the genome of NCIM1228 via homologous recombination. For this, a disruption cassette was designed that had hygromycin resistance gene cassette flanked with 1 kb upstream of *cbh1* ORF at 5′ end and 1.5 kb downstream to *cbh1* ORF at 3′ end (Additional file 1: Fig. S5a). *cbh1* deletion cassette was assembled into pCambia1302 at *Mau*BI/*Xho*I restriction sites to get pCT1 plasmid and transformed into *P. funiculosum* by *Agrobacterium*-mediated method. The transformants were selected on LMP hygromycin agar plates (Additional file 1: Fig. S5a). The transformant colonies were grown from single spores and all transformant colonies appeared were stable for their resistance towards hygromycin after multiple passages. Ten of these transformants were screened by PCR for hygromycin gene integration and *cbh1* gene deletion. Primers corresponding to 300 bp upstream and downstream regions to *cbh1* gene were used for PCR; all the transformants showed amplification of 2.5 kb fragment corresponding to *cbh1* deletion cassette (Additional file 1: Fig. S5a). However, three of these transformants also showed amplification of 2.2 kb fragment corresponding to native *cbh1* gene. This indicated random integration of CBHI deletion cassette leaving native *cbhI* gene intact in the genome. This experiment showed that frequency of homologous recombination of *cbh1* deletion cassette at *cbh1* locus was 7 out of 10 among hygromycin-resistant transformants. Similar deletion strategy was carried out for two other gene loci, *pyr4* (putative gene coding for Orotidine 5′ decarboxylase) and *ku70* (involved in non-homologous end-joining pathway of DNA repair) (Additional file 1: Fig. S5b, c) [16, 17]. *pyr4* and *ku70* deleted transformants were selected in the presence of 5-Fluoroorotic acid (5-FOA) and zeocin, respectively (Additional file 1: Fig. S5b and c). The efficiency of homologous recombination was found to be > 60% among 5-FOA and zeocin-resistant mutants. Interestingly, the length of flanking region and gene locus did not affect the frequency of homologous recombination in NCIM1228 for our tested genes (Table 1). Hence, T-DNA-based DNA cassettes were apt for gene integrations, deletions and mutations in *P. funiculosum* NCIM1228 via *Agrobacterium*-mediated transformation.

**Table 1:** Size of homologous ends used to create deletion cassettes

Gene	Upstream to selection marker cassette (bp)	Downstream to selection marker cassette (bp)	Size of the native gene (bp)
*CBH1*	1109	1614	1566
*pyr4*	500	500	1932
*ku70*	1121	322	2058
*mig1*^*a*^	770	860	1248

^a^Performed in our previous work published in Randhawa et al. [8]

### CRISPR/Cas9 gene editing in *P. funiculosum*

**Fig. 6:**
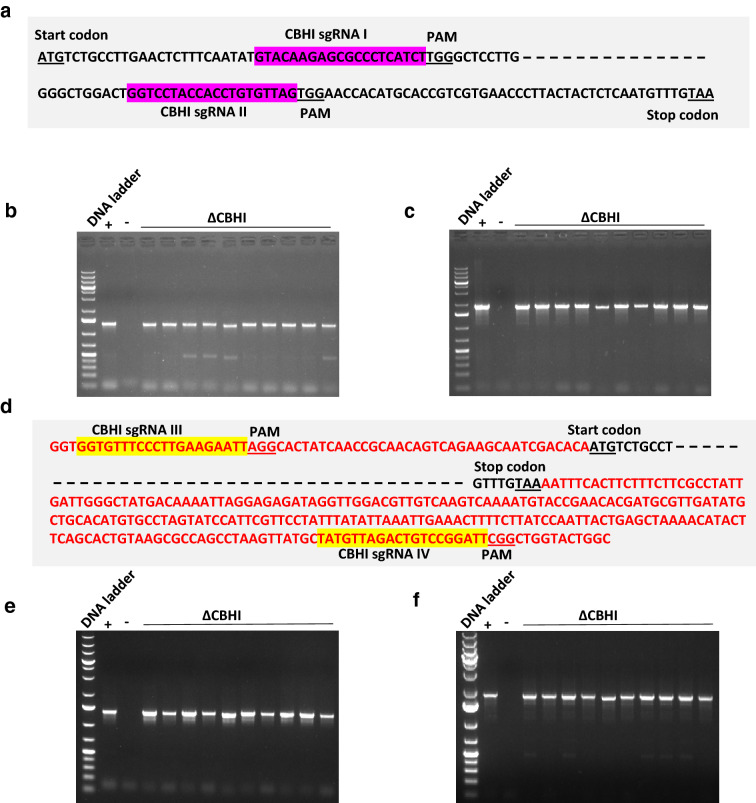
CRISPR/Cas9-based *cbh1* gene deletion in *P. funiculosum* NCIM1228. **a** Schematic representation of *cbh1* ORF to show the position of sgRNA I and II (highlighted in pink) used for complete removal of *cbh1* ORF. 10 hygromycin-resistant transformants were tested for integration of repair template at the target site. Two sets of primers were used; **b** Amplification of 1319 bp fragment amplified by primers CBH1 1 kb up F and *Hph int R*. The amplicon refers to the region 1 kb upstream to *cbh1* ORF and 200 bp of *hph* resistance cassette. **c** Amplification of 1828 bp fragment amplified by primers CBH1 1.5 kb dn R and *Hph int F*. The amplicon refers to the region 1.5 kb downstream to *cbh1* ORF and 200 bp of *hph* resistance cassette. **d** Schematic representation of *cbh1* ORF and flanking region to show the position of sgRNA III and IV (highlighted in yellow) used for complete removal of *cbh1* ORF. 10 hygromycin-resistant transformants were tested for integration of repair template at the target site. **e** Amplification of 1319 bp fragment amplified by primers CBH1 1 kb up F and *Hph int R*. **f** Amplification of 1828 bp fragment amplified by primers CBH1 1.5 kb dn R and *Hph int F*

*Agrobacterium*-mediated method of transformation and genome modification had a high success rate in NCIM1228; however, the method is tedious due to multiple steps of cloning, transformation and selection involved [18]. Recently, *in vitro* CRISPR/Cas9 technology has been shown to function effectively in mammalian cell lines, plants and filamentous fungi [19–21]. Instead of expressing Cas9 and sgRNA in the cell, they are directly introduced into the cell as ribonucleotide protein (RNP) complex [22]. Since it allows precise gene editing with least probability of random mutations, we here attempted to optimize in vitro CRISPR/Cas9 gene editing for *P. funiculosum*. We proposed to delete *cbh1* gene again using this method. Plasmid-free CRISPR/Cas9 method in *P. funiculosum* required Cas9 protein along with nuclear localization signal (Cas9-NLS), sgRNA specific to the target site in genome and repair template having selection marker flanked by homologous ends to the target site. For complete loss of the *cbh1* gene, we designed a pair of sgRNA that would give two double-strand breaks within the *cbh1* ORF, one very close to the start codon and the other close to the stop codon of the *cbh1* ORF (Fig. 6a). The two sgRNAs were in vitro transcribed, purified and made complex with Cas9-NLS to prepare RNP complex. Repair template had hygromycin resistance gene flanked by 300 bp homologous ends upstream and downstream to *cbh1* gene. PEG-mediated fungal transformation was used to introduce RNP complex along with repair template into protoplasts of *P. funiculosum* and transformants were selected in the presence of hygromycin. To rule out random or homologous recombination-based repair template integration in the genome, repair template alone was also used for transformation into fungal protoplasts as control. Only two transformants appeared in the control set, whereas > 100 transformants appeared in case of transformation of *cbh1* deletion RNP complex. Ten transformants from test and two from control transformation plates were inoculated in PD broth and genomic DNA was isolated from grown cultures. All transformants of test group showed PCR amplification of repair template at *cbh1* locus, indicating accurate integration at the desired site (Fig. 6b and c, Additional file 1: S6a). While control transformants did show amplification of the repair template, none of them showed amplification of repair template at the *cbh1* locus (Fig. 6b and c, Additional file 1: S6a). Thus, it appears that double-strand breaks made by the pair of sgRNA might have paved the way for integration of repair template at the correct genome locus.

**Fig. 7:**
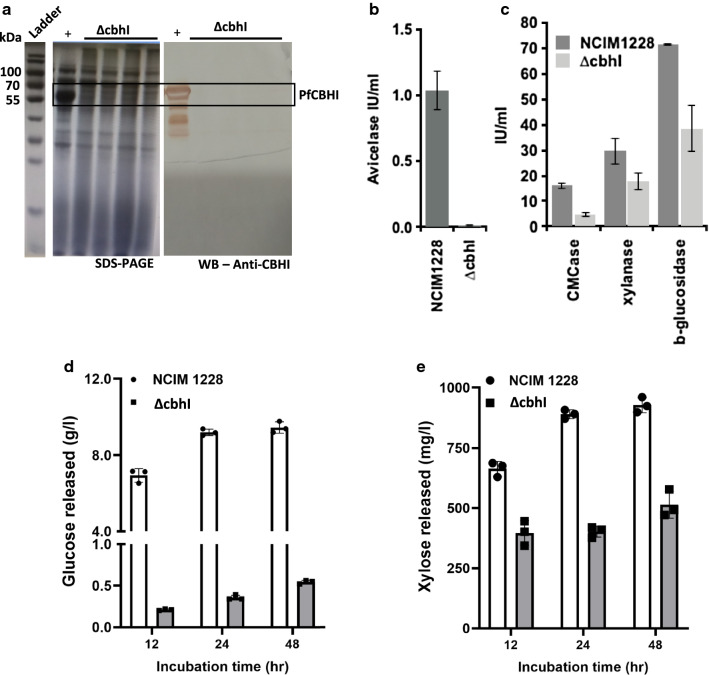
Functional analysis of *Δcbh1* strain secretome. **a** SDS-PAGE gel of secretome of NCIM1228 (+ ve control) and four *Δcbh1* mutants showing band corresponding to CBH1 protein missing in *Δcbh1* secretome; second panel showing Western blot performed with anti-CBH1 antibody confirming the absence of CBH1. **b** Activities for Avicelase **c** CMCase, xylanase and β-glucosidase were measured in the secretome of NCIM1228 and ΔCBH1 when grown in modified RCM cellulase production medium. **d** Glucose and **e** xylose released after time intervals of 12 h, 24 h and 48 h of saccharification of pretreated rice straw (5%) by the NCIM1228 and ΔCBH1 secretome (30 mg/g of dry biomass weight)

It has been reported earlier that the sgRNA site in the repair template cassette increases the frequency of transformation [23]. We thus designed a pair of sgRNAs that were upstream and downstream to the *cbh1* gene and were also present in the repair template (Fig. 7d). The transformation was repeated with new set of *cbh1* sgRNAs along with Cas9 and repair template. 10 transformants were selected and checked for hygromycin resistance cassette integration using the primers from 500 bp upstream and downstream to the *cbh1* locus. All the transformants showed replacement of *cbh1* gene with hygromycin resistance cassette (Fig. 6e, f). We also sequenced four *cbh1* deletion mutants, and while three of them showed expected result, we found random mutations at the sgRNA IV target site downstream to *cbh1* locus in one of the transformants (Additional file 1: Fig. S6b and S6c). While both strategies of plasmid-free CRISPR/Cas9 gene editing system worked in *P. funiculosum*, the first strategy where sgRNA site was absent in repair template had very low error rate. Also, homologous arms up to 300 bp in length efficiently got integrated at the target site. These experiments showed effectiveness of CRISPR/Cas9 gene editing system in *P. funiculosum*, which could be exploited further for genome editing at more than one loci as well as for constructing high-throughput gene deletion libraries.

### *cbh1* deletion mutant lost its ability to hydrolyse crystalline cellulose and pretreated biomass

*cbh1* deletion mutant constructed by CRISPR/Cas9 technology was functionally characterized to study the impact of *cbh1* deletion on the cellulolytic capacity of NCIM1228 and role of other cellulases present in the secretome. Four ∆*cbh1* mutants along with the NCIM1228 were grown in cellulase production media for 5 days. ∆*cbh1 *exhibited poor growth in the cellulase inducing growth medium even after six days of incubation. At the end of 6 days, secretomes from both types of strains were collected by centrifugation and tested for the presence of protein band corresponding to CBH1 by SDS-PAGE (Fig. 7a). In ∆*cbh1 *mutants, band corresponding to CBH1 protein was not found [8, 15]. We also confirmed the absence of CBH1 by Western blotting with anti-CBH1 antibodies (Fig. 7a). We next checked the competency of ∆*cbh1* secretome against NCIM1228 by measuring activities of four major cellulolytic enzymes, namely, exocellulase, endoglucanase, β-glucosidase and xylanase. We found highly reduced Avicelase activity in ∆*cbh1* strain (Fig. 7b). ∆*cbh1* secretome also exhibited reduction in endoglucanase (CMCase) activity by ~ 70% and β-glucosidase (PNPGase) activity by ~ 40% in comparison to NCIM1228 (Fig. 7c). Xylanase activity was also affected in ∆*cbh1* secretome (Fig. 7c). We speculated that reduction in activities of other classes of cellulases might be due to poor growth of ∆*cbh1* mutant strain in cellulase production medium (modified RCM growth medium) and might result in less secretion of proteins, and therefore, we measured the total protein secreted by NCIM1228 and ∆*cbh1* mutants. Since the soluble components of modified RCM growth medium contains organic nitrogen source and usually interfere with protein measurements, we therefore exchanged the growth medium of the secretome of NCIM1228 and ∆*cbh1* mutant with 50 mM Citrate buffer, pH4.0 and measured the total protein secreted by BCA method. In comparison to 4.8 ± 0.23 g/l of protein secreted by NCIM1228, ∆*cbh1* could secrete 3.1 ± 0.05 g/l protein. When we measured the specific activity of four classes of cellulases, we found Avicelase activity was negligible per gramme of secreted protein, whereas specific activity of endoglucanase (CMCase) was reduced by 40% in comparison to secretome of parent strain, NCIM1228 (Table 2). However, marginal decrease was observed in the specific activity of β-glucosidase and xylanase between NCIM1228 and ∆*cbh1* secretome (Table 2). We next examined the effect of ∆*cbh1 *deletion on biomass hydrolysing capability of NCIM1228 secretome. For this, a biomass hydrolysis reaction containing nitric acid pre-treated wheat straw (5% biomass loading) with 30 mg/g of DBW (dry biomass weight) NCIM1228 and ∆*cbh1* secretome was set up at 50 °C and the sugar release at time intervals of 12, 24 and 48 h was measured by HPLC. Wheat straw after nitric acid pre-treatment had 61.3% cellulose and 6% hemicellulose. We found expected glucose release (up to 10 g/L) with NCIM1228 secretome at all the time intervals; however, glucose release by ∆*cbh1* secretome was negligible at early time points and could reach up to 0.5 g/l even after 48 h of incubation (Fig. 7d). We also observed reduction even in xylose release with ∆*cbh1 *secretome; however, the reduction was not as profound as glucose release (Fig. 7e). The above experiments proved the essentiality of CBH1 for cellulose degradation by *P. funiculosum* NCIM1228.

**Table 2:** Specific activities of selected biomass hydrolysing enzymes of Δcbh1 secretome in comparison to *P. funiculosum* NCIM1228

Secretome	Avicelase (U/mg)	CMCase (U/mg)	Xylanase (U/mg)	PNPGase (U/mg)
NCIM1228	0.22 ± 0.03	3.4 ± 0.25	6.2 ± 1.10	14.9 ± 0.09
Δcbh1	0.004 ± 0.001	1.5 ± 0.3	5.7 ± 1.2	12.4 ± 3.0

## Discussion

Transition of a promising fungal isolate into an industrial workhorse involves multiple, regulated rounds of genome editing, rewiring of metabolic and signalling networks, introduction of heterologous genes and pathways, and further validation [24]. Development of molecular and synthetic biology toolbox is a pre-requisite for directed and controlled manipulation of fungal genomes. The present study was conducted to develop molecular tools for *P. funiculosum* NCIM1228 and escalate its capacity to produce superior cellulolytic secretome for second generation bioindustries.

The first issue to address was the phenomenon of drug tolerance exhibited by NCIM1228. Drug tolerance capacitates drug-sensitive micro-organisms to grow slowly at inhibitory concentrations of antibiotics. It differs from drug resistance in being time dependent and is usually exhibited as slow growth after 48 h of incubation in the presence of drug. Tolerance to drugs is independent of drug concentration used and is exhibited by 5–90% of the population size. Since appearance of fungal transformants usually takes 5–7 days, drug tolerance capabilities of *P. funiculosum* proved detrimental for screening the fungal transformants leading to very high cases of false positives. We attempted to understand the probable reason behind the tolerance ability though transcriptomic approach.

Transcriptomic studies conducted under carbon repressing and de-repressing conditions revealed the role of majorly two classes of drug transporter: ATP-Binding Cassette (ABC) superfamily and Major Facilitator Superfamily (MFS) present in NCIM1228. ABC family of transporters are multicomponent energy-driven permeases capable of transporting both small molecules and macromolecules, while the MFS are single-polypeptide carriers involved in transport of small solutes in response to chemiosmotic ion gradients [25]. The biggest fraction of ABC transporter genes were found to code for putative drug efflux pumps in *P. funiculosum* NCIM1228. 70% of the MFS transporters were suggested to be involved in transport of nutrients, vitamins and minerals and rest of the others were proposed to code for drug efflux transporters. When transcriptomic analysis was performed for NCIM1228 grown in simple sugar, i.e. glucose, and complex sugar, i.e. cellulose, it was found that majority of these transporters were being expressed in either or both of the carbon conditions. Our initial hypothesis was that presence of monomeric sugars, like glucose, in the growth medium might induce the fungus to indulge in non-essential but beneficial cellular processes, and one of them could be higher expression of drug efflux pumps. Cellulosic medium, on the other hand, might shift the focus of fungal mycelia away from expressing efflux pumps to produce and secrete cellulase. However, on the contrary, our results indicated greater expression of drug efflux pumps, especially ABC transporters, in the cellulose containing medium. This could be in response to the stress that a complex carbon source might exert on the growth and physiology of the fungus.

An earlier report on *A. niger* had demonstrated the effectiveness of ABC transporter inhibitor, chlorpromazine, along with non-ionic detergent Triton X-100 in combating drug resistance, and we attempted to use these non-selective inhibitors along with antibiotic in growth medium. We also tested the role of different growth media in combating drug tolerance. We found NCIM1228 to exhibit maximum drug tolerance in SC media and, hence, was considered least suitable for selecting transformants. Both PD and LMP agar were found suitable for selection against hygromycin and nourseothricin when accompanied by chlorpromazine and Triton X-100. However, LMP was the best selection medium for absolute sensitivity towards zeocin. Both SC and PDB had 20 g/l glucose as carbon source; however, nitrogen content of the two media varied considerably. SC medium has 1.055 g/l elemental nitrogen in the form of inorganic salt (ammonium sulphate) and PD medium has 0.247 g/l elemental nitrogen majorly in the organic form [26]. These results also suggest the role of nitrogen source in the drug tolerance. Having low levels of carbon and nitrogen might be the reason for LMP growth medium to be the most suitable for selecting transformants. Another interesting observation was distinct morphotype displayed by *P. funiculosum* NCIM1228 on different growth media. Similar phenotype has been observed in *Aspergillus* species and has been suggested to impact its virulence characteristics [27]. Ability of a fungus to change its macroscopic morphotype in response to nutrient availability is quite intriguing. The investigation of molecular changes underlying colony morphotypes will provide insight to understand fungal ways of living and tame them accordingly for industrial and research purposes.

We attempted both PEG/CaCl_2_ and *Agrobacterium*-mediated methods of transformation for NCIM1228. NCIM1228 protoplasts were used for PEG/CaCl_2_-mediated transformation, whereas spores were used for co-cultivation with transformed *Agrobacterium* for *Agrobacterium*-mediated transformation (AMT). When both methods of transformation were tried using optimized selection medium, we could achieve stable transformants with high transformation efficiency solely by AMT. It might be due to the presence of T-DNA repeats providing extra stability to the foreign DNA in the fungal genome [28]. We achieved over-expression of heterologous as well as native gene by random integration. However, the transformants showed varied level of gene expression probably due to the different sites of integration in the genome. Therefore, we suggest screening of large number of transformants to achieve the recombinant strain with high expression of the gene of interest. We have recently used AMT-based random integration to over-express two important cellulases to enhance the cellulolytic capability of NCIM1228 secretome [29].

We next employed *Agrobacterium*-mediated transformation to create gene deletions and mutations. Deletion cassettes were constructed to have selection marker flanked by homologous arms to the targeted gene. These cassettes were cloned within the left and right T-DNA repeats. All the transformants achieved had deletion cassettes integrated into their genome; > 60% of these transformants had deletion cassette integrated at the targeted locus leading to gene deletions/mutations. Further, frequency of homologous recombination was found independent of gene loci as well as lengths of homologous ends in the deletion cassettes. Therefore, the method holds huge potential in creating point mutations, gene truncations, and promoter engineering and replacement in NCIM1228. In our recently published study, we have also used AMT-based transformation along with optimized selection medium to achieve a truncated *mig1* gene in NCIM1228, with precise introduction of stop codon within the ORF, resulting in a truncated Mig1 protein with disrupted zinc finger domain [8]. Out of the two DNA damage repair mechanisms in filamentous fungi, mechanism of non-homologous end-joining (NHEJ) leading to random integrations dominates homologous recombination (HR)-based repair mechanisms, thereby decreasing the frequency of gene targeting by foreign DNA. Therefore, disrupting the KU70/KU80 NHEJ recombination system is a common strategy to increase the frequency of homologous recombination in filamentous fungi. However, high frequency of homologous recombination achieved by *Agrobacterium*-mediated method abolished the need to disrupt the NHEJ recombination system in *P. funiculosum* NCIM1228.

We also developed an RNA-guided CRISPR/Cas9 genome engineering technique for *P. funiculosum.* RNP complex was made *in vitro* and delivered into the fungal protoplasts via PEG-mediated transformation. This approach was promising as it did not require multiple cloning steps of Cas9 and sgRNA cassettes and ensured low probability of off-target editing by sgRNA-Cas9 complex because of its brief presence in the cell. Cellobiohydrolase I was taken as target gene to be deleted and two sgRNAs were constructed complementary to sequences near the start and stop codon of the *cbh1* ORF for complete deletion of the ORF. All the transformants had *cbh1* deleted in their genome and repair template integrated at the targeted locus. Gene deletion was indeed found to be the outcome of double-strand breaks increasing the rate of homologous recombination of repair template at the targeted site. CRISPR/Cas9 technology optimized for *P. funiculosum* NCIM1228 could be a harbinger of rigorous strain engineering and genome-wide functional analysis.

We also functionally characterized the *cbh1* deletion strain. CBH1 constitutes about 15% of the total secretome secreted under inducible conditions and its deletion caused total protein concentration of NCIM1228 secretome to fall by similar magnitude. We examined the effect of *cbh1* deletion on the cellulolytic ability of NCIM1228 secretome. Cellulose degradation is a collaborative action of at least three classes of enzymes; exocellulase (CBH1 and CBH2) hydrolyses ends of crystalline cellulose by releasing mainly a disaccharide at a time, endoglucanase hydrolyses cellulose fibres internally, thereby creating more ends for exocellulase, and β-glucosidase hydrolyses disaccharide into monomeric sugar. We found that CBH1 was largely responsible for exocellulase activity of NCIM128 secretome as its deletion completely abolished the capacity of NCIM1228 secretome to degrade crystalline cellulose. This was surprising, considering *P. funiculosum* NCIM1228 harbours two exocellulases, Cellobiohydrolase I (CBH1) and Cellobiohydrolase II (CBH2), which are the most abundant cellulases expressed under inducing conditions at 10% and 5% of the total identified proteins, respectively [1]. We also found that absence of CBH1 led to 40% decrease in specific activity of endoglucanase (CMCase) as well, while the specific activity of β-glucosidase and xylanase remained unchanged. This possibly suggests that CBH1 of *P. funiculosum* might also utilize the amorphous CMC as substrate in addition to the crystalline Avicel. The activity against CMC was also reported by Santos et al. while characterizing CBH1 of *Penicillium digitatum* [30].

## Conclusion

*Penicillium funiculosum* NCIM1228 being a promising fungal isolate with extraordinary saccharification capabilities, the genetic engineering of this host would play a huge role in its development as industrial host for cellulase production for second generation bioindustries. Thus, the present study was aimed at establishing a genetic tool box for *P. funiculosum* NCIM1228. Firstly, the use of antibiotic selection markers was made possible by resolving drug tolerance issues of the fungus. Next, *Agrobacterium*-mediated transformation was established for gene deletion and replacement in NCIM1228. We achieved a high homologous recombination efficiency using this method without disrupting NHEJ DNA repair pathway. Moreover, a relatively quick method of gene editing was also developed by transforming CRISPR/Cas9 RNP complex into NCIM1228 protoplasts along with DNA repair cassette.

## Materials and methods

### Strains, plasmids, media and growth conditions

*E. coli* DH5α strain was used for cloning and plasmid preparation. The transformants were grown in LB medium supplemented with kanamycin (50 μg/ml) at 37 °C and plasmids were subsequently extracted using Plasmid Miniprep Kit (Qiagen).

For *Agrobacterium*-mediated transformation, *Agrobacterium tumefaciens* LBA4404 strain was used. *Agrobacterium* transformants containing appropriate plasmids were grown in low sodium LB medium supplemented with kanamycin (100 μg/ml) and rifampicin (30 μg/ml) at 30 °C.

The hypercellulolytic fungus *P. funiculosum* NCIM1228 was procured from national collection of industrial microorganisms, NCL, Pune, India and was used as host for genetic manipulation. The growth media for fungus used in this study were potato dextrose medium (HiMedia, India), yeast nitrogen base with ammonium sulphate (Difco) and 2% glucose, and Low malt extract peptone (LMP) media consisting of 1% malt extract (HiMedia, India) and 0.05% Soya peptone (HiMedia, India). Chlorpromazine (0.1 mM) and Triton X-100 (0.01%) were added to the growth media after autoclaving. Hygromycin, nourseothricin and zeocin were added to the media at 50 μg/ml or as specified elsewhere. For comparing growth on agar plates, *P. funiculosum* NCIM1228 and ∆*cbh1* strains were grown at 30 °C for 2, 7 and 14 days. For determining individual enzyme activities, NCIM1228 and Δ*cbh1* strains were inoculated at 10^7^ conidiospores/ml in 50 ml of PD broth, grown for 24 h and 10% of these cultures were used to inoculate secondary modified RCM medium for further 5 days of growth [31].

Binary plasmid pCambia1302 was used for constructing *Agrobacterium*-based deletion cassettes. Complete T-DNA of pCambia1302 was replaced by *P. funiculosum*-specific deletion cassettes. pAN-egfp plasmid having zeocin resistance cassette and *hph* resistance cassette were synthesized commercially (GeneScript Inc) and pBIF-egfp plasmid was constructed in our earlier work [13].

### RNA sequencing and transcriptomic analysis

For sample preparation, NCIM1228 was grown in Mandel Weber media having 1.4 g/l (NH_4_)_2_SO_4_, 0.1 g/l Soya peptone, 2 g/l KH_2_PO_4_, 0.3 g/l CaCl_2_.2H_2_0, 0.3 g/l MgSO_4_.7H_2_O, 5 mg/l FeSO_4_.7H_2_O, 1.6 mg/l MnSO_4_.H_2_O, 1.4 mg/l ZnSO_4_.7H_2_O, 2 mg/l CoCl_2_.6H_2_O, 0.1% Tween-80 and 4% glucose or Avicel. Log phase culture was collected, lyophilized and stored at −80 °C until RNA isolation. Total RNA was isolated from lyophilized samples by Qiagen Plant mini RNeasy kit. RNA was given DNase treatment before proceeding for RNA sequencing. RNA-Seq was performed commercially using the HiSeq 2000 platform with 125 × 2 paired-end read chemistry (Bionivid Technology Pvt Ltd). Biological replicate sequencing libraries for both conditions were created with poly-A tailed mRNA enrichment using the standard Illumina TruSeq mRNA RNA-Seq protocol. Generated Illumina sequencing RNA-Seq reads were assembled using the reference draft genome sequence by using Trinity with genome guided approach. Quantitative levels were generated for all assembled transcripts by mapping all generated sequencing reads to the assembled transcripts using the alignment mapping program Bowtie2 and alignments were coordinate-sorted by SAMtools. All quantitative values were calculated in Fragments Per Kilobase of transcript per Million mapped reads (FPKM) by using quantitative program RSEM. The protein domains were predicted using InterProScan. The ATP-binding cassette (ABC) superfamily transporters and multi-facilitator superfamily (MFS) transporters were filtered and identified from InterPro result. Non-centred heatmaps were generated from log2 FPKM values of selected ABC and MFS genes by performing Hierarchical Clustering using the Euclidean distance matrix option of MeV tool. For differential expression profiling, all FPKM values were normalized to the library size using the R package, Edge R. The obtained p-values were used to assess the significance of transcripts’ up- and downregulation as shown in respective figures.

### Construction of deletion cassettes

*cbh1* deletion cassette: A DNA fragment of 4314 bp having *cbh1* gene along with flanking regions at either side was PCR amplified from genomic DNA using primers CBH1 1 kb up F and CBH1 1.5 kb dn R (Additional file 1: Table S1) and cloned in pCambia1302 replacing T-DNA at *Bst*BI and *Aat*II restriction sites. A *cbh1* deletion construct was generated by removing 1558-bp *cbh1* ORF region from pOAO2 [29] by restriction digestion and replacing it with 1916-bp hygromycin resistance cassette at *Eam*1105I/*Oli*I restriction sites. Chemically synthesized 1916-bp hygromycin cassette (Genscript) was PCR amplified using Hph cas F and Hph cas R primers and cloned at *Eam*1105I/*Oli*I restriction sites. As a result, 12.99 kb binary vector was created having *cbh1* deletion cassette between the T-DNA arms.

*ku70* deletion cassette: A DNA fragment of 2058 bp having *ku70* gene was PCR amplified from genomic DNA using primers ku70 F and ku70 R (Table S1) and cloned in pCambia1302 at *Mau*BI and *Xho*I restriction sites. A *ku70* deletion construct, was generated by removing 627-bp *ku70* ORF region from pKu70 by restriction digestion and replacing it with 1414-bp zeocin resistance cassette at *Hind*III/*Eco*RI restriction sites. The 1414-bp zeocin resistance cassette was PCR amplified from pAN-egfp using zeocin cas F and zeocin cas R primers before cloning at *Hind*III/*Eco*RI restriction sites. As a result, 9275 bp binary vector was created having Ku70 deletion cassette in its T-DNA.

*pyr4* deletion cassette: *pyr4* deletion cassette was prepared by NEBuilder HiFi DNA assembly kit (NEB #E2621). An ORF encoding putative cutinase regulator *ctf1a* (contig id 160_0.0) of 2553 bp was assembled with 500 bp DNA fragments corresponding to upstream and downstream to *pyr4* ORF to form disruption cassette and cloned in pCambia1302 at *Mau*BI and *Xho*I restriction sites, resulting in a binary vector of 10.7 kb size. *ctf1a* gene was earlier found to encode a transcriptional activator for cutinase [32]. It was added in the genome of NCIM1228 to utilize the recombination event to enhance the quality of NCIM1228 secretome towards the plant biomass, which will be characterized in detail in our future study.

### Protoplast-mediated fungal transformation

Protoplast preparation: The fungal spores were inoculated in 25 ml PDB and incubated for 36 h at 30 °C. Mycelia were then harvested, suspended in protoplast buffer (1.2 M MgSO_4_, 10 mM Sodium Phosphate pH 7.0) having *Trichoderma harzianum* cellulase (Sigma) and incubated for 3 h. Filtrate collected after the incubation was overlaid with separation buffer (0.6 M sorbitol, 100 mM Tris–Cl pH 7.0) and centrifuged for 1500*g for 15 min. The protoplasts ring formed between the separation and protoplasts buffer was collected separately and the protoplasts were pelleted by centrifugation at 3000*g for 10 min. The pellet was resuspended in 500 µl of STC/PEG (50% Polyethylene glycol (MW 3350), 10 mM CaCl_2_, 10 mM Tris–Cl pH7.5 and 1.2 M Sorbitol) and viewed under microscope at 100X magnification. Protoplasts were counted using haemocytometer at 20X magnification and 10^7^ protoplasts were used for transformation [33].

PEG/CaCl_2_-based protoplast transformation: PEG-mediated fungal transformation was achieved as described before with minor modifications [33]. 5 µg of linearized pAN-egfp was added to 10^7^ protoplasts along with 50% polyethylene glycol (PEG) (MW 3350) and incubated for 20 min. The transformants were selected in the presence of zeocin and the colonies appeared after five (or more) days of incubation. Transformants were transferred to fresh selection plates and passaged for 3–4 times to determine the stability.

### *Agrobacterium*-mediated fungal transformation

Preparation of *A. tumefaciens* chemical competent cells: *A. tumefaciens* LBA4404 was grown overnight in 10 ml LB-Lennox broth (5 g/l NaCl, 5 g/l yeast extract, trptone 10 g/l) having 30 µg/ml rifampicin overnight at 30 °C. Grown culture (1%) was used to inoculate 100 ml LB-Lennox broth having 30 µg/ml rifampicin. Culture was collected at OD_600_ of 0.3–0.5 and chilled on ice for 30 min with regular shaking. Cells were harvested by centrifuged at 4000 rpm for 10 min at 4 °C, washed thrice with ice-cold CaCl_2_ buffer (100 mM of CaCl_2_ and 10% glycerol) and resuspended in 2–3 ml of ice-cold CaCl_2_ buffer. The cells were aliquoted in 1.5-ml microcentrifuge tubes (100 µl/tube), snap frozen in liquid N_2_ and stored at −70 °C.

Transformation of *A. tumefaciens* by heat shock method: 1–2 µg of binary vectors were added to chemical competent cells and the cells were incubated on ice for an hour. Cells were given 5 cycles of heat shock at 37 °C for 30 s with interim incubation on ice for 30 s. The cells were transferred to 10-ml falcon tube having 2 ml of low sodium LB broth and were incubated at 30 °C with shaking for 4 h. Cells were collected by centrifugation and plated on low sodium agar with 50 µg/ml kanamycin and 30 µg/ml rifampicin. Plates were incubated at 30 °C and colonies appeared within 48–72 h.

*Agrobacterium*-mediated transformation of *P. funiculosum*: Freshly grown spores of *P. funiculosum* NCIM1228 were used as fungal recipient and *A. tumefaciens* LBA4404 carrying appropriate binary vector served as donor for *Agrobacterium*-mediated fungal transformation as described earlier [34]. Briefly, freshly transformed *Agrobacterium* was pre-induced for T-DNA mobilization in induction medium [34]. Pre-induced *Agrobacterium* cells at OD_600_ of 0.3 were mixed with fungal spore suspension at 1:1 in induction medium (IM) and plated on cellophane discs overlaid on IM agar plates supplemented with 200 µM acetosyringone. Plates were incubated at 22 °C for 48 h until the appearance of thin mycelial layer on top of the cellophane discs. Cellophane sheets were then transferred to selection plates having 200 μM cefotaxime and appropriate antibiotic. Transformants appeared after 5–14 days of incubation at 30 °C. PfCBH1, PfKu70 and PfPyr4 deletion transformants were selected for hygromycin, zeocin and 5-FOA resistance, respectively. Gene deletion was confirmed by amplification of newly acquired disruption construct in place of coding region with flanking 5′ and 3′ regions.

### CRISPR/Cas9-based gene editing of *P. funiculosum* NCIM1228

sgRNA-Cas9 (RNP) complex preparation: sgRNAcas9 software package was used to generate sgRNA sequences for *cbh1* gene [35]. The two sgRNAs for complexing with Cas9-NLS were synthesized using the EnGen sgRNA synthesis kit available from New England Biolabs (NEB #E3322) according to the instructions provided and purified before complexing to Cas9 using the RNA clean & Concentrator-25 Kit (Zymo Research). Equimolar ratio of Cas9-NLS (NEB #M0646T) and sgRNA were mixed to have sgRNA-Cas9 complex and incubated at room temperature for 20 min.

DNA repair template for CRISPR/Cas9-based gene deletion: DNA repair template was generated from *cbh1* deletion cassette binary vector. Primers, CBH1 300 bp up F and CBH1 300 bp up R, were used to amplify hygromycin cassette along with 300 bp homologous arms of CBH1. The PCR product was used as repair template in RNP-CRISPR generated double-stranded break (DSB).

Transformation of RNP complex and repair template: sgRNA-Cas9 complex was transformed into NCIM1228 protoplasts by PEG/CaCl_2_-based transformation method as described above. The RNP complexes and the repair template (10 µg) were added to the protoplasts along with 50% polyethylene glycol (PEG) (MW3350) and incubated for 20 min. Transformants were selected in the presence of hygromycin. Stable hygromycin-resistant transformants were screened for *cbh1* deletion by PCR using two pairs of primers. The first pair of primers annealed to upstream of *cbh1* promoter (CBH1 1 kb up F) and internal region of hygromycin gene (Hph IR R), while the second pair annealed to internal region of hygromycin gene (Hph IR F) and downstream of *cbh1* terminator (CBH1 1.5 kb dn R).

### Southern hybridization

Southern hybridization was performed using standard procedures described by Southern [36]. 8 µg of Genomic DNA of NCIM1228 or pBIF-egfp transformants was digested with *Bam*HI and size-fractionated by electrophoresis on 0.8% agarose gel in 1 × TAE (Tris–acetate) buffer. After three steps, namely, depurination (using 250 mM HCl), denaturation (using 1.5 M NaCl and 0.5 M NaOH) and neutralization (using 1.5 M NaCl and 1.0 M Tris–HCl pH 8.0), the gel was capillary blotted onto a positively charged Hybond™-N + membranes (Amersham Biosciences, USA). The 717-bp of *egfp* ORF was PCR amplified with the primers GFP F and GFP R and labelled with [α-^32^P] dCTP using the NEBlot Kit (NEB, USA) and was used as a probe to detect pBIF-egfp integration. Similarly, 500-bp of hph ORF was PCR amplified with primers Hyg IR F and Hyg IR R and labelled with [α-^32^P] dCTP to be used as probe to detect pBIF-CBH1 integration.

### Protein estimation, SDS-PAGE, Western blotting and enzyme assays

Secretomes of NCIM1228 and ∆*cbh1* were obtained by centrifuging the culture at 8000 rpm for 10 min. Protein estimation of secretomes was done by BCA (Bicinchoninic acid) kit (G Biosciences). Equal volume of secretome was loaded on 10% SDS-PAGE gel and total protein was visualized by staining the gel with Coomassie blue. CBH1 expression in the secretome of NCIM1228 and ∆*cbh1* was detected by Western blotting using rabbit anti-CBH1 polyclonal antibodies and mouse anti-rabbit HP-conjugated secondary antibody (Cell Signaling Technology) as described in our earlier study [8, 31]. The activities of secretome enzymes towards 0.5% Avicel, 1% CMC and 1% beech-wood xylan were measured using the dinitrosalicylic acid (DNSA) method as described in our earlier study [8, 31]. β-glucosidase activity was determined by measuring the amount of *p*-nitrophenol released from *p*-nitrophenyl-β-d-glucopyranoside (pNPG) [8, 31].

### Biomass hydrolysis of nitric acid pre-treated wheat straw

Saccharification potential of the NCIM1228 and ∆*cbh1 *secretome towards nitric acid pretreated rice straw was determined according to the method described by Ogunyewo et al. [31]. Saccharification reaction was set up in 1.5-mL microcentrifuge tubes in an incubator shaker at 50 °C for 12, 24 and 48 h. The reaction mixture included the pretreated biomass at 5% dry weight loading and secretome was added at a protein concentration of 30 mg/g of dry weight of biomass in a 250 μL of final reaction volume. The product released upon hydrolysis were analysed using HPLC.

## Supplementary information

**Additional file 1:** **Table S1.** List of primers used in this study. **Table S2.** Determination of minimal inhibitory concentration of antibiotics for *P. funiculosum* NCIM1228. **Fig. S1.** Susceptibility of *P. funiculosum* NCIM1228 towards hygromycin, zeocin and nourseothricin. **Fig. S2.** Sensitivity of *P. funiculosum* NCIM1228 towards antibiotics on PD and LMP agar supplemented with Triton X-100 and chlorpromazine after 14 days of incubation. **Fig. S3.** Sensitivity of *P. funiculosum* NCIM1228 towards antibiotics on LMP agar supplemented with chlorpromazine, Triton X-100 and both after 7 days of incubation. **Fig. S4.** Random integration of cassette in the genome of *P. funiculosum.* **Fig. S5.** Deletion of *cbh1*, *ku70* and *pyr4* genes in *P. funiculosum* NCIM1228. **Fig. S6.** Genetic characterization of *cbhI* deletion transformants.

## Data Availability

The data and materials used in the current study will be made available from the corresponding author on reasonable request.
