# Supplementary material for: Blocking drug efflux mechanisms facilitate genome engineering process in hypercellulolytic fungus, Penicillium funiculosum NCIM1228
Source: Biotechnol Biofuels. 2021 Jan 25;14:31. doi: 10.1186/s13068-021-01883-4 (PMC7836482; doi:10.1186/s13068-021-01883-4)
Supplement: Supplementary file 1 — Additional file 1: Table S1. List of primers used in this study. Table S2. Determination of minimal inhibitory concentration of antibiotics for P. funiculosum NCIM1228. Fig. S1. Susceptibility of P. funiculosum NCIM1228 towards hygromycin, zeocin and nourseothricin. Fig. S2. Sensitivity of P. funiculosum NCIM1228 towards antibiotics on PD and LMP agar supplemented with Triton X-100 and chlorpromazine after 14 days of incubation. Fig. S3. Sensitivity of P. funiculosum NCIM1228 towards antibiotics on LMP agar supplemented with chlorpromazine, Triton X-100 and both after 7 days of incubation. Fig. S4. Random integration of cassette in the genome of P. funiculosum. Fig. S5. Deletion of cbh1, ku70 and pyr4 genes in P. funiculosum NCIM1228. Fig. S6. Genetic characterization of cbhI deletion transformants. [file 13068_2021_1883_MOESM1_ESM.pdf]

Supplementary Information for

"Blocking drug efflux mechanisms facilitate genome engineering process in hypercellulolytic fungus *Penicillium funiculosum* NCIM1228"

Supplementary Table S1. List of primers used in the study

Supplementary Table S2. Determination of minimal inhibitory concentration of antibiotics for *P. funiculosum* NCIM1228.

Supplementary Fig. S1 Susceptibility of *P. funiculosum* NCIM1228 towards hygromycin, zeocin and nourseothricin.

Supplementary Fig. S2 Sensitivity of *P. funiculosum* NCIM1228 towards antibiotics on PD and LMP agar supplemented with Triton X-100 and chlorpromazine after 14 days of incubation.

Supplementary Fig. S3 Sensitivity of *P. funiculosum* NCIM1228 towards antibiotics on LMP agar supplemented with chlorpromazine, Triton X-100 and both after 7 days of incubation.

Supplementary Fig. S4 Random integration of cassette in the genome of *P. funiculosum.*

Supplementary Fig. S5 Deletion of *cbh1, ku70* and *pyr4* genes in *P. funiculosum* NCIM1228.

Supplementary Fig. S6 Genetic characterization of *cbhI* deletion transformants.

Supplementary Table S1. List of primers used in the study

Primer Name	Primer Sequence (5’-3’)
EGFP F	ATGGTGAGCAAGGGCGAG
EGFP R	TTACTTGTACAGCTCGTCCATG
pBIF hph IR F	AGATCGTTATGTTTATCGGCAC
pBIF hph IR R	GCTGTTATGCGGCCATTGTC
hyg_IR_F	AGATCGTTATGTTTATCGGCAC
CBHI_R_IR	ATCTAACGTAATGGCACCGG
1440_CBHI_F	TAGTTCGTCAGCGTCAACTG
CBHI_IR_R	GGTCACGAAGTAGAGGGCA
CBHI 1kb up	CATCTTCGAATATCCCAACAGGTCGATCC
CBHI 1.5 kb dn R	GAGATACTCGGATTGACGTCGCGC
CBHI 300bp up F	GGACGTATAGATCAGGGACTGTGAGG
CBHI 300bp up R	TACGGCTTGAGATGGAATTTTGGGC
Hph cas F	AATCGACACAATGTCCTGCAGTAATCAATCC
Hph cas R	GGTTCACGACGGTGAACCACTAGTACGCGTGGATCCGTC
Hph int R	TTCTCAAGCTTGGATTCACCTCAC
Hph int F	TTAGCTCATTCAAGACTTTATTTACGCC
Zeocin cas F	CAGAAGCTTCTGATTTAATAGCTCCATGTC
Zeocin cas R	GACTGAATTCCATCTGTAGGGCGTCC
Ku70 F	TAACGCCGAATTAATATGGCTGACAGTAACCCCC
Ku70 R	ACACCGCGCGCGTTAACCCTTTCGGTCAAAATAC
PyrG 500bp up F	acgcTCTAGACGCCCACGGCAATCTAGTCG
PyrG 500bp dn R	ACGCCGCGCGCGTACAAAGTGCTATCCCTATCAATGAAATTAC CAG
PyrG seq F	CCGAATGGTCTTGGACGG
PyrG seq R	GACTGTCAATGTAAACAAGTTGATGTGGG

Supplementary Table S2. Determination of minimal inhibitory concentration of antibiotics for *P. funiculosum* NCIM1228. Number of NCIM1228 colonies appeared after 10^4 spores were plated on different concentrations of antibiotics after 48 hours of incubation.

	**25 µg/ml**	**50 µg/ml**	**75 µg/ml**	**100 µg/ml**
**Hygromycin**	**128±34**	**47±5**	**0**	**0**
**Zeocin**	**96±27**	**44±14**	**0**	**0**
**Nouroseothricin**	**21±6**	**0**	**0**	**0**

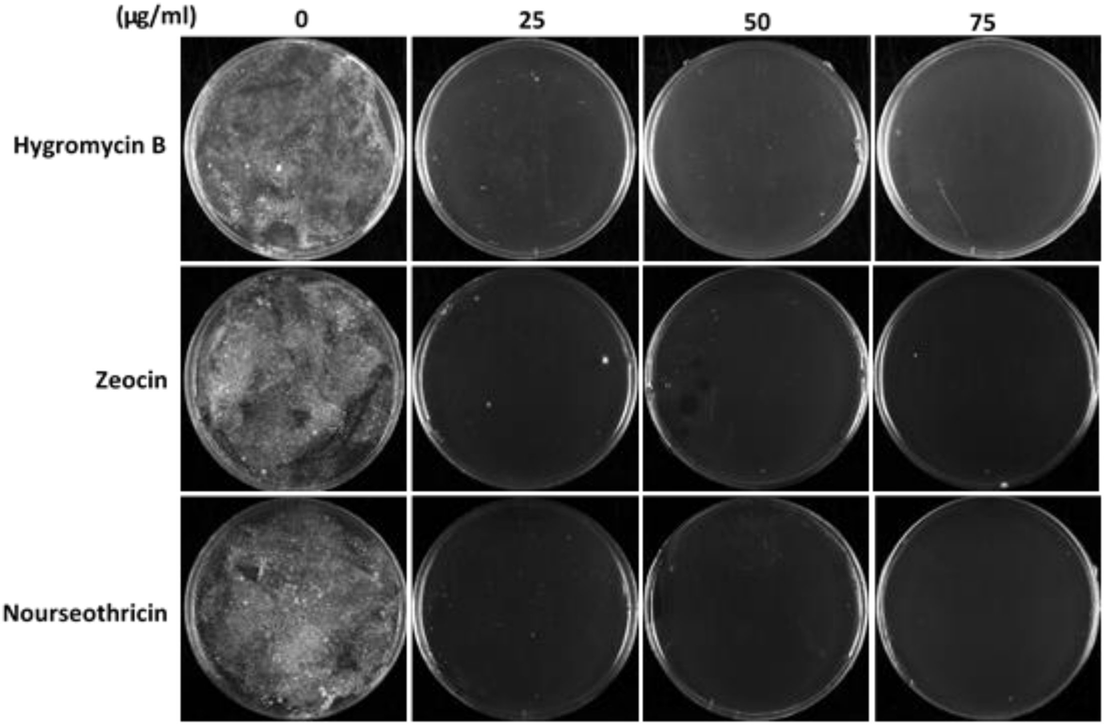

Supplementary Fig. S1 Susceptibility of *P. funiculosum* NCIM1228 towards hygromycin, zeocin and nourseothricin. 10^4 spores were plated on PD agar plates having antibiotics ranging from 25 to 100 µg/ml. Complete inhibition of NCIM1228 was found at 75 µg/ml of hygromycin and zeocin and 50 µg/ml of nourseothricin after 48 hours of incubation.

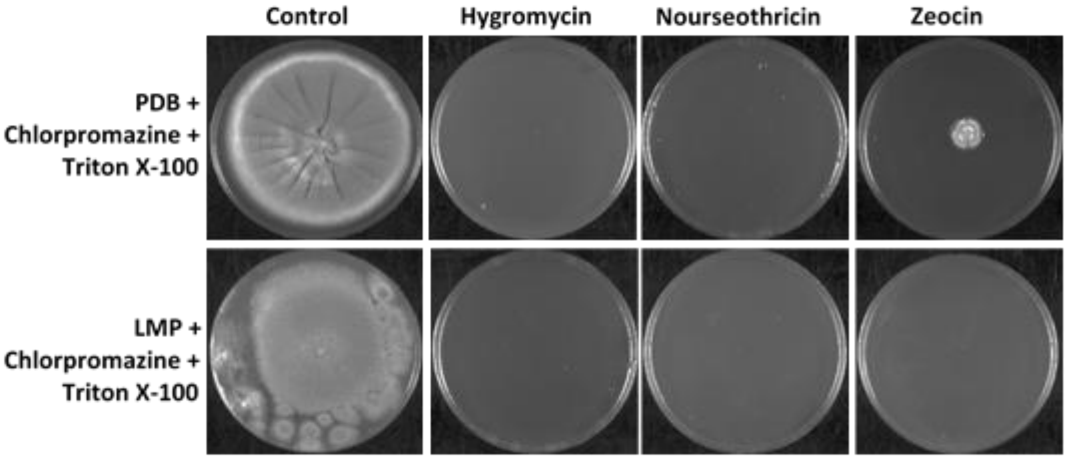

Supplementary Fig. S2 Sensitivity of *P. funiculosum* NCIM1228 towards antibiotics on PD and LMP agar supplemented with Triton X-100 and chlorpromazine after 14 days of incubation.

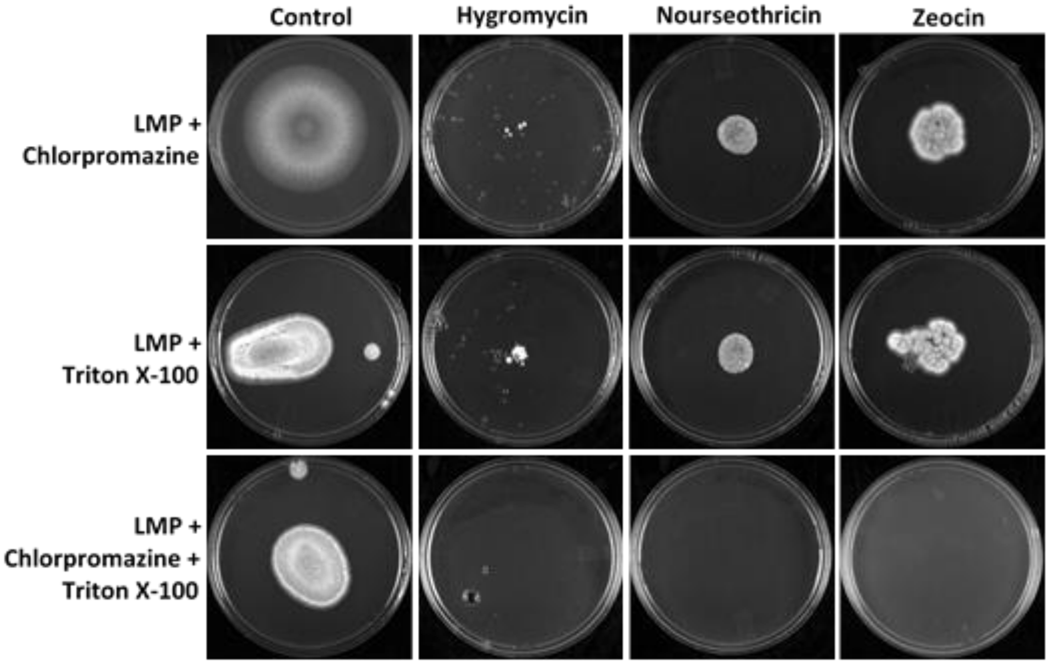

Supplementary Fig. S3 Sensitivity of *P. funiculosum* NCIM1228 towards antibiotics on LMP agar supplemented with chlorpromazine, Triton X-100 and both after 7 days of incubation.

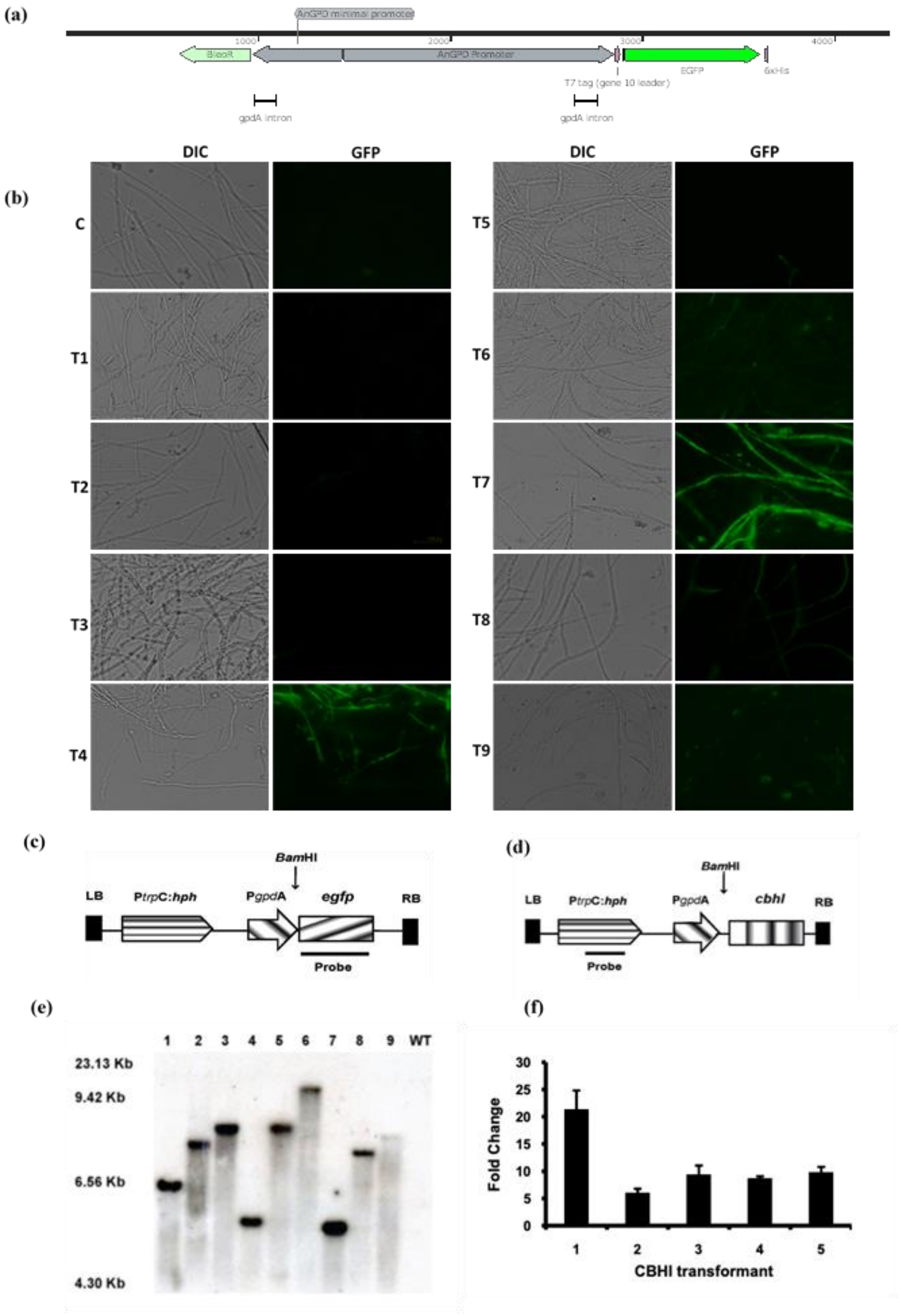
(a)
AnGPD minimal promoter
1000
2000
3000
4000
BleoR
AnGPD Promoter
EGFP
6xHis
T7 tag (gene 10 leader)
gpdA intron
gpdA intron
(b)
DIC
GFP
DIC
GFP
C
T1
T2
T3
T4
T5
T6
T7
T8
T9
(c)
BamHI
LB
PtrpC:hph
PgpdA
egfp
RB
Probe
(d)
BamHI
LB
PtrpC:hph
PgpdA
cbhI
RB
Probe
(e)
1 2 3 4 5 6 7 8 9 WT
23.13 Kb
9.42 Kb
6.56 Kb
4.30 Kb
(f)
Fold Change
30
25
20
15
10
5
0
1 2 3 4 5
CBHI transformant

Supplementary Fig. S4 Random integration of cassette in the genome of *P. funiculosum* (a) Schematic representation of pAN-egfp expression cassette used for protoplast LiAc-PEG based transformation in *P. funiculosum* NCIM1228. (b) Transformants of randomly integrated pBIF-EGFP show varied levels of expression. Schematic representation of (c) pBIF-EGFP and (d) pBIF-cbh1 to show the probes used for southern blots and position of BamHI restriction enzyme used for generating DNA fragments. (e) Southern blot of pBIF-cbh1 transformants showing integration of T-DNA having CBHI gene. A 460 bp fragment from hygromycin resistance cassette was used as probe. NCIM1228 (WT) was taken as negative control. (f) The transcriptional expression of cbhI in pBIF-cbh1 transformants were measured by quantitative real-time PCR after growing for 24 h in the presence of glucose. The expression levels were normalized to NCIM1228 and plotted.

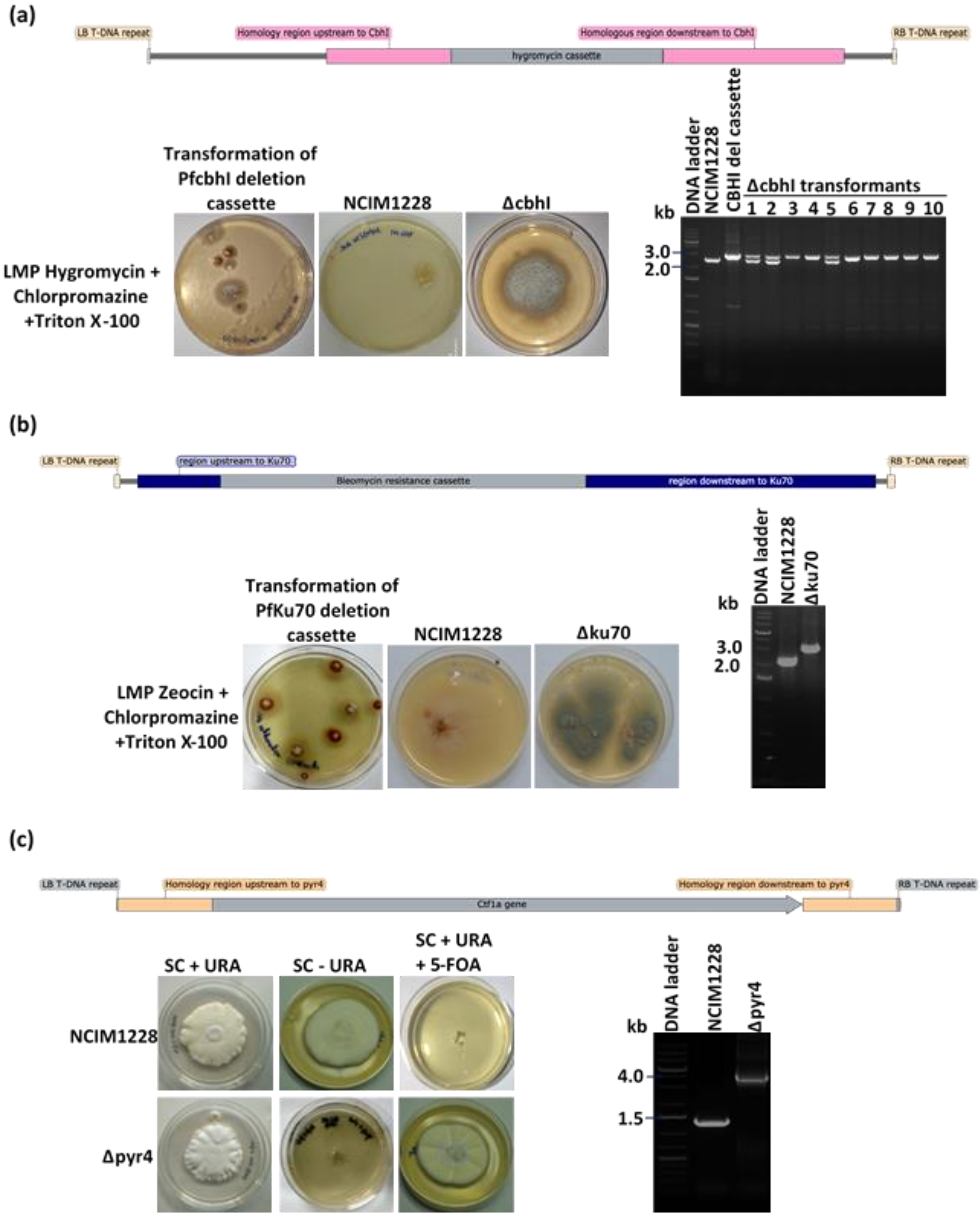
(a)
LB T-DNA repeat
Homology region upstream to CbhI
hygromycin cassette
Homologous region downstream to CbhI
RB T-DNA repeat
Transformation of PfcbhI deletion cassette
NCIM1228
ΔcbhI
LMP Hygromycin + Chlorpromazine +Triton X-100
DNA ladder
NCIM1228
CBHI del cassette
ΔcbhI transformants
1 2 3 4 5 6 7 8 9 10
kb
3.0
2.0
(b)
LB T-DNA repeat
region upstream to Ku70
Bleomycin resistance cassette
region downstream to Ku70
RB T-DNA repeat
Transformation of PfKu70 deletion cassette
NCIM1228
Δku70
LMP Zeocin + Chlorpromazine +Triton X-100
DNA ladder
NCIM1228
Δku70
kb
3.0
2.0
(c)
LB T-DNA repeat
Homology region upstream to pyr4
Ctf1a gene
Homology region downstream to pyr4
RB T-DNA repeat
SC + URA
SC - URA
SC + URA + 5-FOA
NCIM1228
Δpyr4
DNA ladder
NCIM1228
Δpyr4
kb
4.0
1.5

Supplementary Fig. S5 Deletion of *cbh1, ku70* and *pyr4* genes in *P. funiculosum* NCIM1228. (a) Schematic representation of *cbh1* deletion cassette transformed. Left panel shows the transformation of *cbh1* deletion cassette via *Agrobacterium*-mediated method and appearance of hygromycin resistant transformants. Single spores from transformed colonies were grown on LMP hygromycin plates to achieve pure transformed colonies. Right panel shows amplification of *cbh1* deletion cassette in hygromycin resistant transformants. Primers corresponding to 300bp upstream and downstream to *cbh1* gene were used for amplification. As expected, NCIM1228 showed amplification of 2166 bp fragment (1566 bp cbhI ORF + 600 bp flanking regions) whereas cbhI deletion strains showed amplification of 2516 bp fragment (1916 *hph* resistance gene cassette + 600 bp flanking regions). Three of the transformants also showed amplification for both native *cbh1* gene as well as *cbh1* deletion cassette suggesting random integration and therefore, intact cbhI gene in these transformants. (b) Schematic representation of *ku70* deletion cassette transformed. Left panel shows the transformation of *ku70* deletion cassette and appearance of zeocin resistant transformants. Right panel shows amplification of native *ku70* gene (2058 bp) from NCIM1228 and *ku70* deletion cassette (2860 bp) from one of the transformants showing using ku70 forwards and reverse primers. (c) Schematic representation of *pyr4* deletion cassette transformed. Left panel shows the transformation of *pyr4* deletion cassette via *Agrobacterium*-mediated method and 5-FOA resistant transformants which showed no growth in the absence of uracil. Right panel shows amplification of native *pyr4* gene (1894 bp) from NCIM1228 and *pyr4* deletion cassette (4090 bp) from one of the transformants using primers for region 500bp upstream and downstream to *pyr4* gene.

(a)

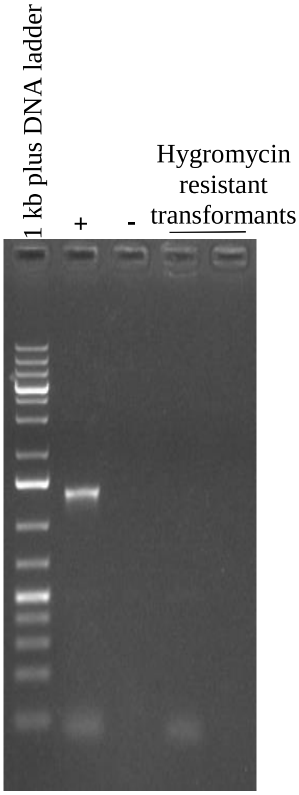

(b)

```
S10     ACTGAGCTAAAACATACTTCAGCACTGTAAGCGCCAGCCTAA------------------
REF     ACTGAGCTAAAACATACTTCAGCACTGTAAGCGCCAGCCTAAGTTATGCTATGTTAGACT
        ******************************************
                                                     cbhI sgRNA IV

S10     -----GATTCGGCTGGTACTGGCTTTTGTGATCCCCAGTATCATGTAAAGGTATCCGCCT
REF     GTCCGGATTCGGCTGGTACTGGCTTTTGTGATCCCCAGTATCATGTAAAGGTATCCGCCT
             *******************************************************
                 PAM
```

(c)

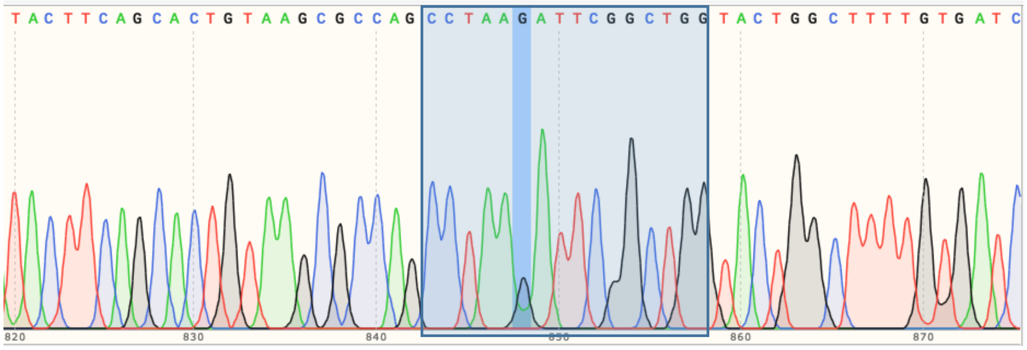

Supplementary Fig. S6 Genetic characterization of *cbhI* deletion transformants. (a) Two Hygromycin resistant transformants appeared in control experiment when NCIM1228 protoplasts were transformed only with repair template. None of them showed amplification of 1319 bp fragment amplified by primers cbhI 1kb up F and *Hph int R*. The amplicon refers to the region 1kb upstream to cbhI ORF and 200bp of *hph* resistance cassette. Repair template was amplified as positive control and NCIM1228 was taken as negative control. (b) Sequence alignment of

reference *cbh1* gene sequence (ref) of *P. funiculosum* NCIM1228 and S10 is the sequence of one of the cbhI deletion mutant which showed mutation at the sgRNA site after DNA repair (c) Mutation found in one of the transformants near the sgRNA IV site when sgRNA external to *cbh1* ORF were used for deleting *cbh1*. The boxed nucleotides show the new junction formed after the deletion of sgRNA and some extra nucleotides.
